# Flexible Carbon Nanotubes Confined Yolk-Shelled Silicon-Based Anode with Superior Conductivity for Lithium Storage

**DOI:** 10.3390/nano11030699

**Published:** 2021-03-11

**Authors:** Na Han, Jianjiang Li, Xuechen Wang, Chuanlong Zhang, Gang Liu, Xiaohua Li, Jing Qu, Zhi Peng, Xiaoyi Zhu, Lei Zhang

**Affiliations:** 1 School of Material Science and Engineering, School of Environmental Science and Engineering, Chemical Experimental Teaching Center, School of Automation, Qingdao University, No. 308, Ningxia Road, Qingdao 266071, China; 2018020395@qdu.edu.cn (N.H.); jjli@qdu.edu.cn (J.L.); 2018020384@qdu.edu.cn (X.W.); 2018205858@qdu.edu.cn (C.Z.); 2019025785@qdu.edu.cn (G.L.); 2019020442@qdu.edu.cn (X.L.); 2017201339@qdu.edu.cn (J.Q.); pengzhi@qdu.edu.cn (Z.P.); 2 Key Laboratory of Materials Physics, and Anhui Key Laboratory of Nanomaterials and Nanotechnology, Institute of Solid State Physics, Chinese Academy of Sciences, Hefei 230031, China

**Keywords:** silicon, yolk−shell structure, anode, lithium-ion batteries

## Abstract

The further deployment of silicon-based anode materials is hindered by their poor rate and cycling abilities due to the inferior electrical conductivity and large volumetric changes. Herein, we report a silicon/carbon nanotube (Si/CNT) composite made of an externally grown flexible carbon nanotube (CNT) network to confine inner multiple Silicon (Si) nanoparticles (Si NPs). The in situ generated outer CNTs networks, not only accommodate the large volume changes of inside Si NPs but also to provide fast electronic/ionic diffusion pathways, resulting in a significantly improved cycling stability and rate performance. This Si/CNT composite demonstrated outstanding cycling performance, with 912.8 mAh g^−1^ maintained after 100 cycles at 100 mA g^−1^, and excellent rate ability of 650 mAh g^−1^ at 1 A g^−1^ after 1000 cycles. Furthermore, the facial and scalable preparation method created in this work will make this new Si-based anode material promising for practical application in the next generation Li-ion batteries.

## 1. Introduction

Silicon (Si) is the most promising anode candidate in lithium-ion batteries (LIBs) due to its high theoretical specific capacity (~4200 mAh g^−1^) and cut-price [1,2,3,4]. However, the large volume changes (over 400% expansion after full lithiation) induced poor structural stability and continuous breaking and regenerating of the solid-electrolyte interphase (SEI) cause’s short working life for Si-based anodes [5,6,7]. Moreover, the low electrical conductivity of the Si limits its rate performance under high current densities [8,9,10]. Up till now, introducing a reserved void space and conductive framework into silicon-based materials has been regarded as the most effective strategy to fundamentally improve the electrochemical behavior of Si-based anodes [11,12,13]. The introduced reserved space can buffer the huge changes in volume of Si during cycling, leading to the enhanced structural integrity and cycling stability [14,15,16]. Additionally, the conductive framework within the composites increases the overall conductivity of the electrodes, resulting in the high-rate capacities under high current densities [17,18,19,20].

Among various Si-based composites, the yolk-shelled Si/carbon (Si/C) composites are the most promising candidate because of their distinctive advantages over the existing Si-based composites in terms of cycling stability and rate behavior [21,22,23,24]. Many previous reports confirmed the effective structure [25,26,27]. For these yolk-shelled Si/C composites, the Si-yolk was encapsulated within a hollow C-shell with reserved space between the Si-yolk and the C-shell. Therefore, the volume changes of inner Si-yolk can be accommodated by the void space and confined within the hollow C-shell, leading to increased structural stability and limited formation of the outer generated SEI film [25,26,27]. However, the introduced void space limits the conductive contact between Si-yolk and C-shell and further decreases the tap density of the composite [28,29,30]. Carbon nanotubes (CNTs) with excellent mechanical properties and high electrical conductivity are regarded as another hopeful carbon matrix to increase the overall behavior of Si-based materials [31,32,33,34,35,36]. Currently, most of the reported Si/CNT anodes are synthesized by directly using expensive commercialized CNTs to mix with Si nanoparticles (Si NPs), causing increased production cost [37,38,39]. Moreover, it is difficult to achieve the uniform distribution between CNTs and Si NPs due to their large surface area [40,41]. Currently, new Si/CNT anodes composites have been developed via a chemical vapor deposition (CVD) process, which provides distinguished structural stability and electrochemical performance, enhances the overall conductivity of the electrode, and increases the safety of the battery [32,42]. Moreover, it is remaining a great challenge to prepare promising Si/CNT composites with low-cost methods while preserving the unique volume change containment functionality of Si/C yolk–shell structures.

**Scheme 1:**
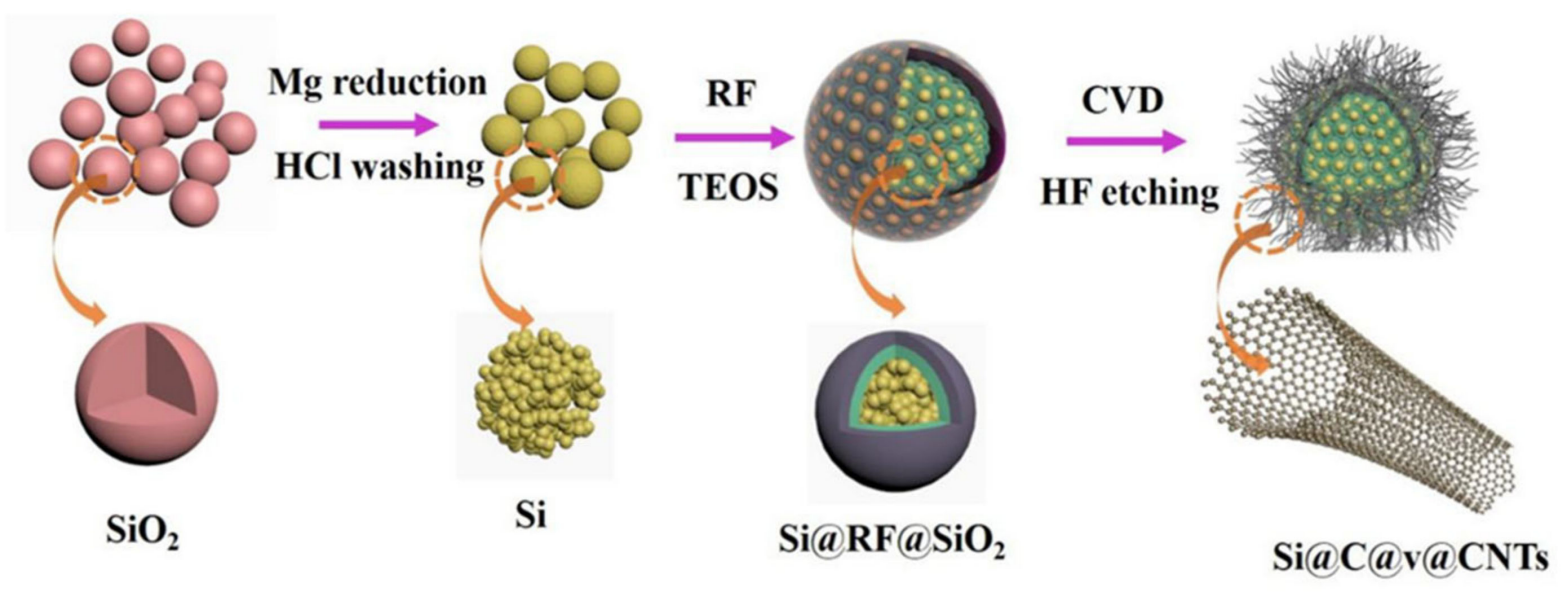
Schematic illustration of the preparation of the Si@C@v@CNTs.

Herein, we overcome these obstacles by developing new Si/CNTs anodes (Scheme 1). Si NPs were successively double-coated with rigid carbon and silica layers (Si@C@SiO_2_) to better encapsulate the incorporated multiple Si NPs to realize good safety levels. Furthermore, the SiO_2_ coating layer on the outer surface of Si@C@SiO_2_ further provided active position for in situ CNTs grown via a CVD method, resulting in a new Si/CNT composite. For this new Si/CNT, the flexible CNT networks were grown on the surface of Si@C@SiO_2_ particles. Therefore, the aggregation for both the CNTs and Si NPs can be significantly suppressed due to the external in-situ grown CNTs networks. Additionally, compared with the traditional yolk-shell structure, the CNT networks and the carbon coating shell effectively increase the conductive contact, not only between the inner Si-yolks and CNT networks but also among different Si/CNT microparticles, leading to increased electronic conductivity and rate capacities. Moreover, the overall structural stability and integrity of this new Si/CNT can also be enhanced by flexible porous CNT networks and rigid carbon coating [42].

## 2. Materials and Methods

### 2.1. Synthesis of Si NPs

All reagents in this paper were purchased from Sinopharm Co (Shanghai, China). The nano-sized silica (SiO_2_) spheres were firstly synthesized by the well-established Stöber method. In the following magnesiothermic reduction (MR) process, Mg powders (99%) and the obtained SiO_2_ spheres were uniformly mixed and placed in one side of a crucible boat. After that, a certain amount of NaCl (AR) (SiO_2_:NaCl = 1:10) was placed in the other side of the crucible boat. The crucible boat was then placed in the center of the tube furnace (OTF-1200X, Shenzhen kejing-zhida Co, Shenzhen, China) and increased to 700 °C under an Ar/H_2_ (95:5 vol. %) flow and retained for 6 h. After cooling down to normal condition, the obtained sample was dispersed in 1 M HCl for several hours to remove NaCl and byproduct MgO. The final porous Si NPs powders were obtained after a wash and vacuum dry.

### 2.2. Synthesis of Si@RF@SiO_2_

The above prepared Si NPs were modified with 3-aminopro-pyltrimethoxysilane (APTES) (AR) to positively charge the surfaces. In total, 0.4 g Si NPs were uniformly dispersed in 300 mL ethanol (AR), containing 4 mL of APTES, and stirred for 5 h to obtain APTES-Si NPs. The above APTES-Si NPs was re-dispersed in an alkaline mixture of 150 mL deionized water and 30 mL ethanol, containing 1 mL of aqueous ammonia (AR) under magnetic stirring for 30 min. After that, 0.6 g resorcinol (AR) and 0.8 mL formaldehyde (AR) were separately added to the reaction system and continued stirring for 10 h to form a homogeneous phenolic resin (RF) coating layer under room temperature. The Si@RF powders were obtained via centrifugation treatment of the reaction solution. For the SiO_2_-coated Si@RF composite (named Si@RF@SiO_2_), 400 mg Si@RF was mixed with 1.5 mL tetraethyl orthosilicate (TEOS) (AR) hydrolysis under alkaline condition to form SiO_2_ coating layer on the outer surface of Si@RF.

### 2.3. Synthesis of Si@C@v@CNTs 

Carbon nanotubes were grown in situ via a CVD method using Iron(III) nitrate nonahydrate (AR) as the catalyst (Fe) and acetylene (5%) as carbon precursors at 900 °C for 2 h under Ar/H_2_ in a tube furnace. The catalyst was loaded on the precursor microspheres of Si@RF@SiO_2_ prior to the deposition procedure to ensure that the CNTs could be grown in situ on the active positions during the CVD process. Finally, the SiO_2_ coating layer was removed with dilute hydrofluoric acid (AR) solution and followed by centrifugation treatment and ethanol washing. After removing the SiO_2_ sacrificial coating layers, the final composite was named Si@C@v@CNTs (“v” stands for “void”). For comparison purposes, the Si@C@v@C (“v” stands for “void”) without in-situ grown CNTs was also synthesized via the same CVD method but in the absence of a Fe(NO_3_)_3_·9H_2_O catalyst, and the Si@v@CNT without an inner carbon layer was also synthesized via the same CVD method in the absence of the RF-layer.

### 2.4. Characterizations

The XRD patterns of samples were obtained via a DX-2007 X-ray diffraction (XRD) experiment apparatus (λ = 1.5418 Å) (Dandong Haoyuan Instrument Co, Dandong, China) to confirm the phases and crystallinity. The Si content of composites was characterized by a thermogravimetric analyzer (TGA4000) (NSK LTD, Tokyo, Japan) at a heating rate of 10 °C min^−1^ in air atmosphere. The nitrogen adsorption/desorption isotherm curves were obtained via a Micromeritics ASAP-2020M nitrogen adsorption/desorption apparatus (Best Instrument Technology Co, Beijing, China) to confirm the porosity character. The micro-structures and morphologies of the materials were collected via a JSM-6700F scanning electron microscope (SEM) (JEOL, Tokyo, Japan) with an IE300X energy-dispersive X-ray spectrometer and a JEM-2100F transmission electron microscope (TEM) (JEOL, Tokyo, Japan). X-ray photoelectron spectroscopy (XPS) curves were obtained by applying an electron spectrometer (ESCALab250) (Thermo Fay, Boston, MA, USA) to analyze the surface of composites. Raman spectra was collected with a Raman spectrometer (JobinYvon HR800) (Renishaw, London, UK). 

### 2.5. Electrochemical Measurements

The CR2016 coin-type cells were assembled in the glove box under inert atmosphere conditions without water and oxygen to test the electrode performance by using polypropylene films as separators (the thickness of the separator was 25 µm) to separate the working and counter electrodes (lithium wafer) in an electrolyte. The electrolyte was the 1 M LiPF_6_ dissolved in the solvent of ethylene carbonate and dimethyl carbonate (the amount of the electrolyte used in assembling the coin-type cell was 70 µL). The working electrode was made by coating the slurry of the above active materials containing a proportional conductive carbon black and polyvinylidene fluoride (PVDF) binder on the copper foil (the mass loading of the electrode was about 1.2 mg cm^−2^), which was then dried under a 120 °C vacuum oven for 14 h (the thickness of the active electrode layer was about 20 µm). Finally, the copper foil was cut into wafers with a uniform size of 1 cm. The galvanostatic cycling measurements were conducted by a CT 2001A battery tester at determinate voltage windows. Cyclic voltammogram (CV) tests were performed by using an electrochemical workstation within a fixed voltage range and scan rate.

## 3. Results and Discussions

**Figure 1:**
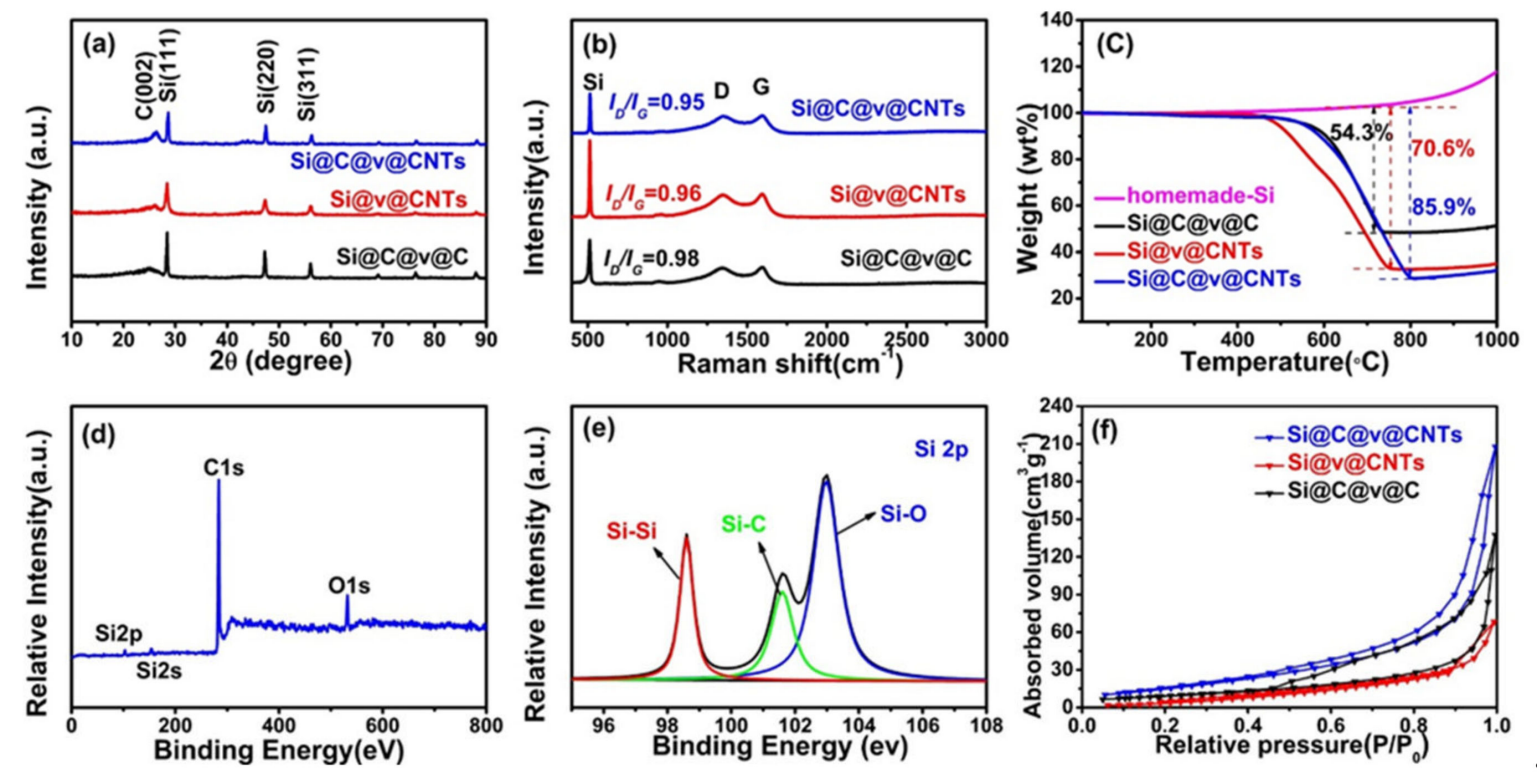
XRD patterns (**a**), Raman spectra (**b**), and thermogravimetric analysis (TGA) curves (**c**) of Si@C@v@C, Si@v@CNTs, and Si@C@v@CNTs; X-ray photoelectron spectroscopy (XPS) of Si@C@v@CNTs (**d**), High-resolution XPS spectrum of Si 2p (**e**), and N_2_ adsorption-desorption isotherms curves of Si@C@v@C, Si@v@CNTs, and Si@C@v@CNTs (**f**).

Figure S1 shows the XRD patterns of reduced Si NPs, Si@RF, and Si@RF@SiO_2_. Three sharp diffraction peaks, which are located at 2θ values of 28.4, 47.2, and 56.1°, were attributed to the planes of (111), (220), and (311) for the crystal Si phase, respectively (JCPDS NO. 27-1402), indicating that the amorphous SiO_2_ synthesized by the well-established Stöber method were fully reduced to crystalline Si in the MR process [43]. Another broad peak at ∼25° corresponded with the amorphous carbon and silica coming from the double coat with RF and silica layers. As shown in Figure 1a, for Si@C@v@CNTs and Si@v@CNTs, after the CVD process, CNT grown in situ across Si-CNPs and the diffraction peak at ∼25° was observed, corresponding to the (002) plane of the crystalline carbon [40]. Figure 1b displays the Raman spectra of Si@C@v@CNTs, Si@v@CNTs, and Si@C@v@C. The sharp peak at about 500 cm^−1^ could be appointed to the Si peaks. Additionally, the weak peaks at about 1345 cm^−1^ (D-band) and 1595 cm^−1^ (G-band) could be associated with the vibration modes of sp^3^-bonded carbon atoms in amorphous carbon and sp^2^-bonded carbon atoms in typical graphite, respectively [44]. As calculated, the *I_D_/I_G_* was 0.98, 0.96, 0.95 for Si@C@v@C, Si@v@CNTs, and Si@C@v@CNTs, respectively. Si@C@v@CNTs had a relatively higher graphitization degree due to the microcrystalline structure of CNTs. Thermogravimetric analysis (TGA) was tested to confirm the proportion of carbon and silicon for the samples (Figure 1c). The weight losses occurred from 500 to 800 °C were ascribed to the carbon combustion and calculated to be 54.3%, 70.6%, and 85.9% for Si@C@v@C, Si@v@CNTs, and Si@C@v@CNTs, respectively. As the temperature continued to rise, Si NPs were further oxidized, leading to a weight increase in the TGA curves. The Si content in the three samples were 45.7%, 29.4%, and 14.1% for Si@C@v@C, Si@v@CNTs, and Si@C@v@CNTs, respectively. Compared with the other two samples, high C and CNT content in Si@C@v@CNTs provided more electric contact between particles, thus enhanced overall electronic conductivity and superior performance could be expected. The elemental compositions and valence states in the composites were determined by X-ray photoelectron spectroscopy (XPS) spectra. Figure 1d reveals the whole spectrum of Si@C@v@CNTs, confirming the coexistence of Si, C, and O. The high-resolution spectrum of Si 2p is shown in Figure 1e. Three peaks of Si-Si (98.6 eV), Si-C (101.6 eV), and Si-O (102.98 eV) originated from monatomic Si, residual silica, or slight oxidation of the Si NPs [45]. In addition, the high-resolution C 1s and O 1s in Figure S2 shows that the peak at 283.75 eV was related to the graphite-like sp^2^ hybridized carbon and another peak at 283.3 eV was assigned to C-O. The O 1s peak at 537 eV may be attributed to adsorbed oxygen and the residual silica layer [46].

The Brunauer−Emmett−Teller (BET) specific surface area and pore volume for the samples are illustrated in Table S1. Compared with Si@C@v@C, the Si@C@v@CNTs had a relatively higher BET specific surface area of 106.98 m^2^ g^−1^, with a pore volume of 0.3212 cm^3^ g^−1^. We believe that the higher surface area was mainly due to the existence of reserved void space and in-situ grown CNT networks. The nitrogen adsorption–desorption isotherm curve of the samples is displayed in Figure 1f. All the curves had a distinct hysteresis loop, suggesting the existence of a mesoporous structure [47]. The pore size distribution curve (Figure S3) shows a diverse pore structure between 2 and 10 nm, which incorporate a series of micro- and mesopores derived from CNT networks [48]. Furthermore, these multiple pore structures not only afford fast and shortened electronic/ionic diffusion pathways but also absorb the huge expansion in volume inside Si NPs during cycling, resulting in a significantly enhanced overall structural integrity and electrochemical performance.

**Figure 2:**
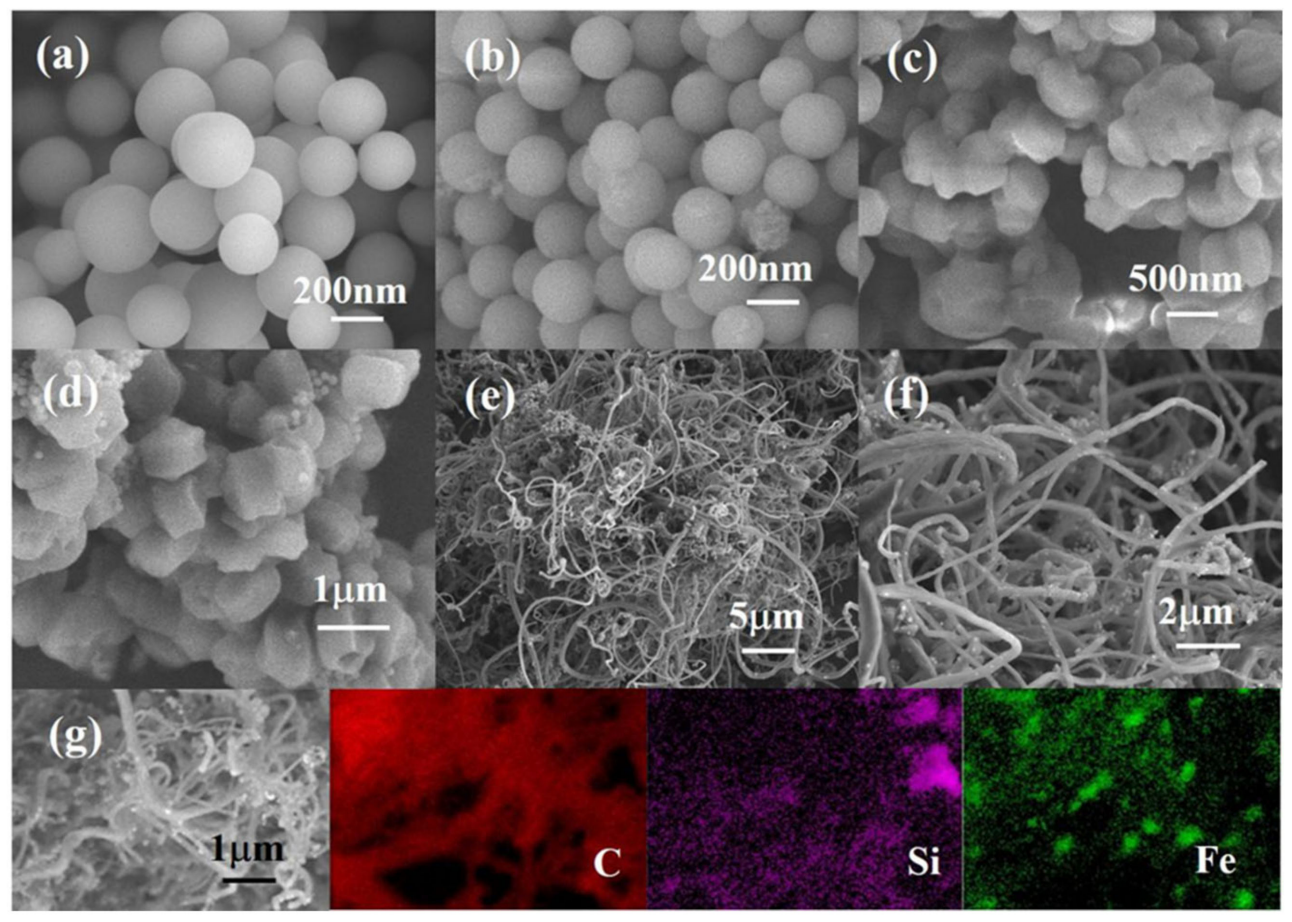
SEM images of SiO_2_ nanoparticles (NPs), synthesized by the well-established Stöber method (**a**), reduced Si NPs in the magnesiothermic reduction (MR) process (**b**), SEM images of the in-process samples: Si@RF (**c**), Si@RF@SiO_2_ (**d**), Si@C@v@CNTs (**e**,**f**). (**g**) the elemental mapping results of Si@C@v@CNTs.

Figure 2 shows the scanning electron microscopy (SEM) images of the structural evolution of Si@C@v@CNTs at different synthesis stages. The SEM image of SiO_2_ spheres firstly synthesized by the well-established Stöber method is shown in Figure 2a. The pristine SiO_2_ spheres present a monodispersed spherical shape with a uniform diameter of approximately 300 nm. After the Mg-reduction process, porous Si NPs with well-preserved monodispersed spherical morphology were successfully prepared (Figure 2b). Figure 2c,d show the SEM images of Si@RF and Si@RF@SiO_2_. The diameters of as-prepared Si@RF and Si@RF@SiO_2_ precursors were increased to 400 nm and 500 nm, respectively, indicating the successful coating of RF and SiO_2_ layers on the Si cores, resulting in Si@C@SiO_2_ particles. Figure 2e,f reveals a large amount of tangled CNTs with various diameters in the range of 50–100 nm externally grown in situ across the Si@C@SiO_2_ particles. The multiple Si NPs were well supported by the in-situ generated flexible porous CNTs networks, contributing to better electric contact, not only between the inner Si-yolks but also among the Si-CNT microparticles. Figure 2g reveals the uniform elemental distribution of the Si, C, and Fe in Si@C@v@CNTs. It is clearly observed the Si-yolks were well coated by the outer C-shell and distributed across the flexible CNT networks. Figure S4 shows the SEM images of Si@C@v@C. The observed wrinkles over the entire surface confirm the full encapsulation of Si@C@SiO_2_ by the carbon layer. The SEM images of Si@v@CNTs in Figure S5 reveal the similar tangled CNTs with Si@C@v@CNTs.

**Figure 3:**
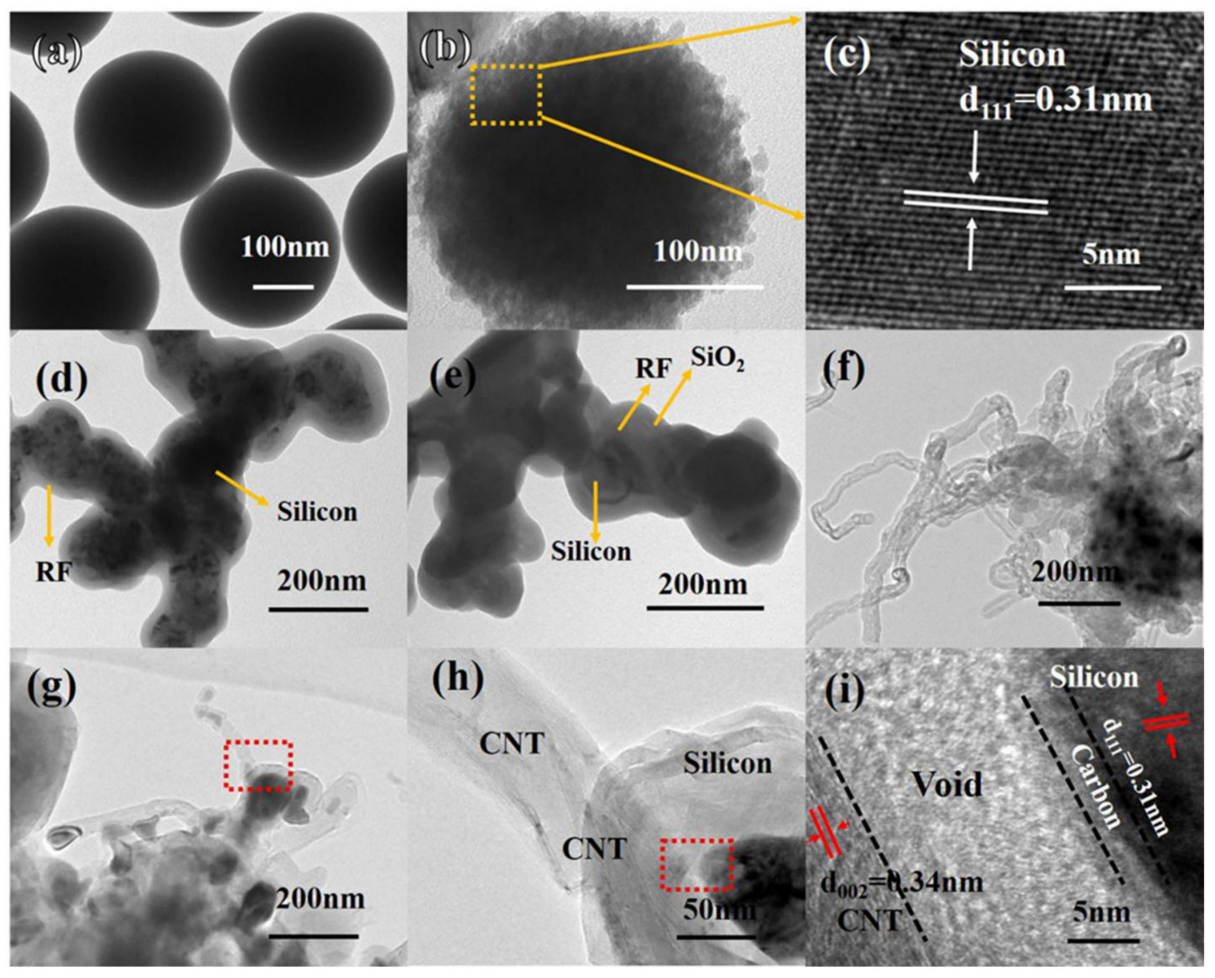
Transmission electron microscope (TEM) images of SiO_2_ NPs synthesized by the well-established Stöber method (**a**), reduced Si NPs in the MR process (**b**), high resolution TEM (HRTEM) images of Si NPs (**c**), TEM images of Si@RF (**d**), Si@RF@SiO_2_ (**e**), and Si@C@v@CNTs (**f**–**h**), and HRTEM images of Si@C@v@CNTs (**i**).

Figure 3a–c shows the transmission electron microscopy (TEM) images of homemade SiO_2_ and reduced Si NPs. The high-resolution TEM image (HRTEM), taken from one of the Si NPs, reveals that the obtained Si NPs had high crystallinity [49,50]. The TEM images of Si@RF and Si@RF@SiO_2_, as shown in Figure 3d–e, confirm that the Si NPs were well wrapped by the RF carbon-SiO_2_ double coating layers. The TEM and HRTEM images of Si@C@v@CNTs presented in Figure 3f–i confirm CNTs grown in situ on the outer layer of the Si@RF NPs with a void between the two. The carbon layer derived from RF was about 5 nm and could accelerate electron transfer between Si NPs and CNTs, leading to enhanced structural stability. The void generated due to the etching of SiO_2_ could effectively alleviate the expansion of the inner Si NPs. According to the TEM images of a single CNT shown in Figure S6, the outer diameter of this single CNT was approximately 35 nm and the tube wall was 12 nm. The TEM image of Si@C@v@C in Figure S7 shows that the Si NPs were well wrapped with double carbon layers. The TEM images of Si@v@CNTs in Figure S8 confirms that existence of similar CNTs grown to Si@C@v@CNTs but without of a carbon layer on the Si NPs.

**Figure 4:**
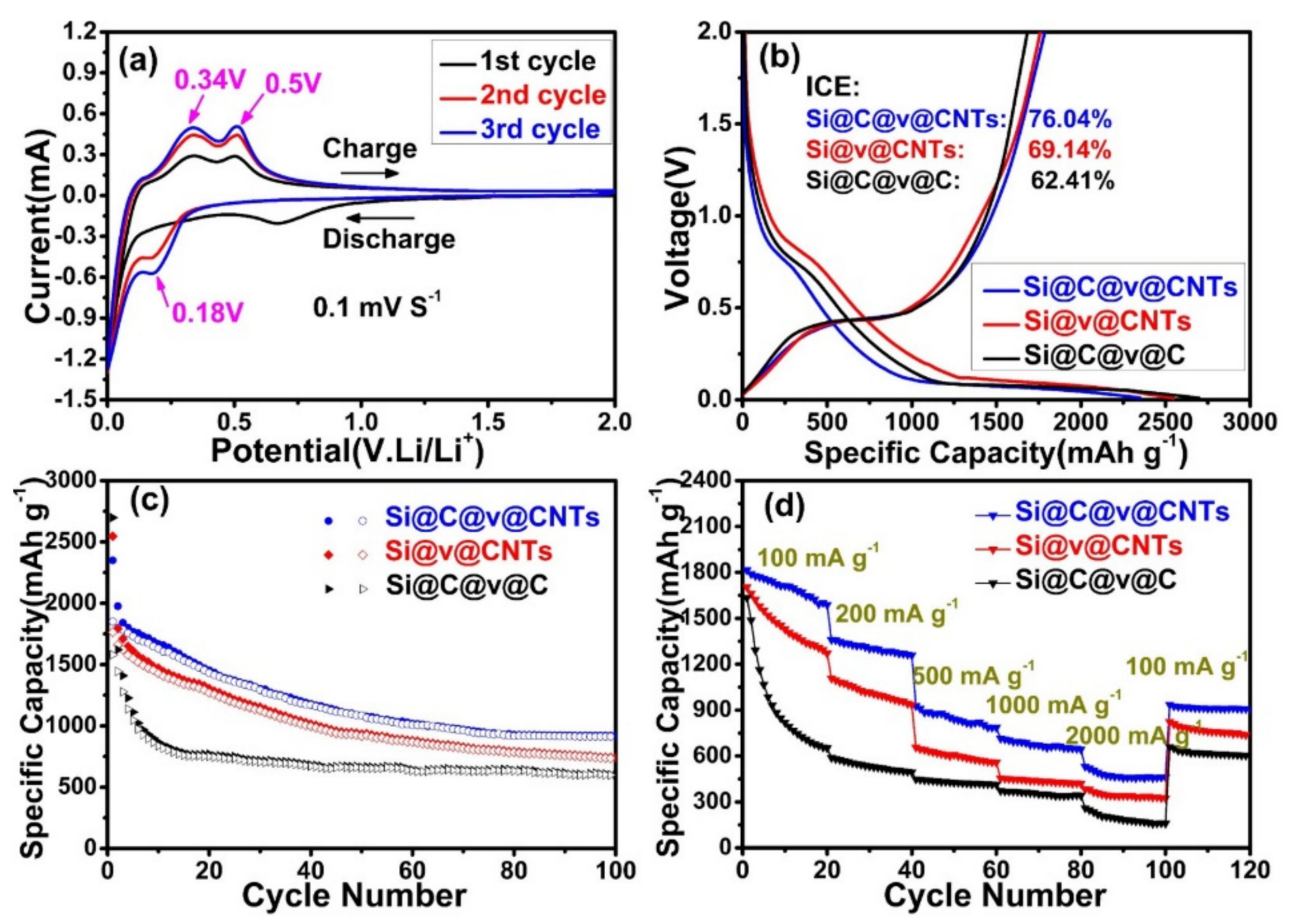
Electrochemical properties: cyclic voltammetry for the first three cycles of Si@C@v@CNTs at a scan rate of 0.1 mV s^−1^ between 0–2.0 V (**a**), the initial discharge–charge curves at 100 mA g^−^^1^ (**b**), cycling performances at 100 mA g^−^^1^ (**c**), the rate performances (**d**) of Si@C@v@C, Si@v@CNTs, and Si@C@v@CNTs.

The cyclic voltammetry (CV) curve of Si@C@v@CNTs exhibited the typical electrochemical properties of Si-based anode materials (as shown in Figure 4a). In the first cathodic branch (lithiation), a distinct broad peak between 0.5 and 0.8 V was ascribed to the generated solid-electrolyte interphase (SEI) film. However, this peak disappeared after the first lithiation, suggesting the generation of the firm and stable films during the first cycle [51]. The lithiation peak at 0.18 V can be appointed to the lithiation process of Si. In the following anodic branch (delithiation), the peaks located at 0.34 and 0.5 V were ascribed to the dealloying process from Li_x_Si to amorphous Si [52]. Figure 4b shows the initial charge–discharge profiles of the materials at 100 mA g^−1^. The disappearance of the voltage plateaus between 0.5 and 0.8 V after the first cycle also confirms the generation of stable SEI films, which is in accordance with the CV results in Figure 4a [44]. The initial discharge and charge capacities were 2698.4 and 1684.2 mAh g^−1^ for Si@C@v@C, 2546.5 and 1760.6 mAh g^−1^ for Si@v@CNTs, and 2350.1 and 1787.0 mAh g^−1^ for Si@C@v@CNTs at 100 mA g^−1^, corresponding to the initial coulombic efficiencies (ICE) of 62.41, 69.14, and 76.04%, respectively. Figure S9 shows the galvanostatic charge–discharge curves of Si@C@v@CNTs during the first five cycles at 100 mA g^−1^. After the first cycle, the CE increased to 92.73% in the following cycling test and reached 96.04% after the fifth cycle. Most importantly, the voltage plateaus during the cycling test was well-maintained, suggesting improved electrochemical utilization of the active electrode materials. The cycling behavior in Figure 4c shows the charge capacities at 100 mA g^−1^ for Si@C@v@C.

**Figure 5:**
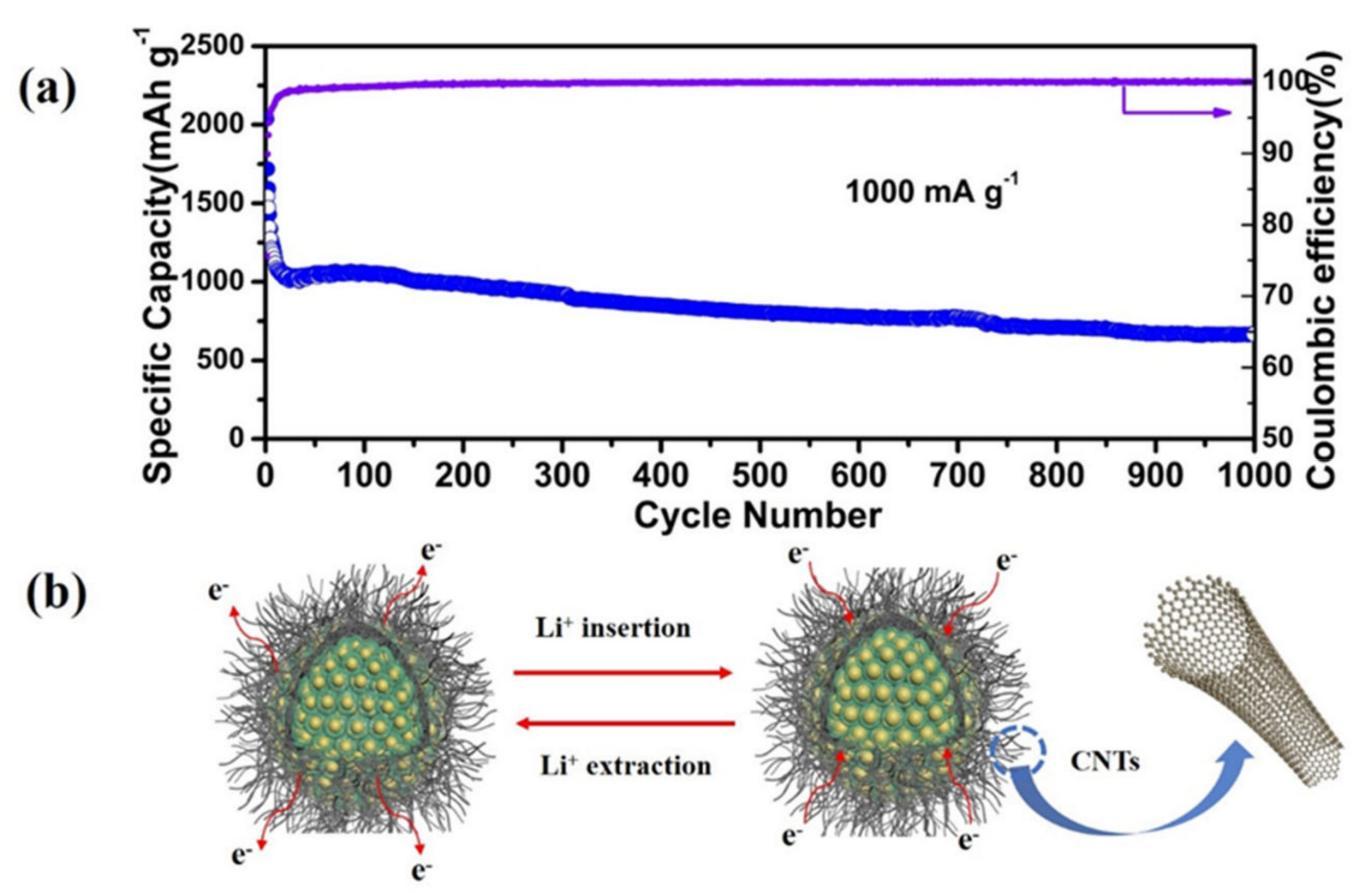
Cycle performance of Si@C@v@CNTs at a current density of 1000 mA g^−1^ (**a**) and a schematic illustration of lithiation and delithiation processes (**b**).

Si@v@CNTs and Si@C@v@CNTs were 598.7, 736.1, and 912.8 mAh g^−1^ after 100 cycles, respectively, indicating that the Si@C@v@CNTs were endowed with the best cycling behavior. Therefore, the outer in-situ grown CNT networks can significantly improve the cycling behavior and structural integrity of the inside-coated Si NP anodes. Figure 4d shows the rate performances of Si@C@v@C, Si@v@CNTs, and Si@C@v@CNTs performed at a series of different current densities. As expected, Si@C@v@CNTs present the best rate ability, even at high current densities. Very high reversible capacity of 907.7 mAh g^−1^ was maintained when the current density was back to 100 mA g^−1^, suggesting an excellent rate ability of Si@C@v@CNTs. Furthermore, a high reversible capacity of 650 mAh g^−1^ was retained for Si@C@v@CNTs at high 1 A g^−1^ after 1000 cycles (Figure 5a). Therefore, it can be concluded that the introduced void space and porous CNT networks can absorb the huge volume expansion inside Si NPs, leading to enhanced overall structural stability and integrity [52,53,54]. In addition, the CNT networks and inner rigid carbon coating provided more sufficient conductive contact to fast electronic/ionic diffusion pathways, resulting in significantly improved cycling stability and rate performance [55,56,57]. Figure S10 shows the SEM images of Si@C@v@CNTs after 1000 cycles at 1 A g^−1^. No cracking can be detected for Si@C@v@CNTs, and all the active materials were still well adhered to the current collector without exfoliation (Figure S10a,b). In addition, the morphology was well maintained in Si@C@v@CNTs after the long cycling test. (Figure S10c). Figure 5b shows the schematic illustration of lithiation and delithiation processes.

Moreover, we fabricated a full cell with our Si@C@v@CNTs as the anode and LiNi_0.6_Co_0.2_Mn_0.2_O_2_ (NCM622) as the cathode (Si@C@v@CNTs//NCM622). The voltage profiles of NCM622 are shown in Figure S11. The NCM622 cathode exhibited a stable reversible capacity of 160 mAh g^−1^ with a flat charging/discharging plateau at about 3.5 V. Referring to the voltage profiles of the Si@C@v@CNTs anode and the NCM622 cathode from half cells, the working potential range for the full cell was set between 2 and 4 V. The cycling performance of Si@C@v@CNTs//NCM622 is shown in Figure S12. The full cell displays a reversible capacity of 92 mAh g^−1^ at 100 mA g^−1^ after 100 cycles, indicating potential cycling stability for commercial viability.

## 4. Conclusions

In summary, we synthesized a yolk-shelled structured silicon/carbon nanotube composite for high performance lithium storage application. This novel Si-based anode was made of an external grown flexible CNT network to confine the inner multiple Si NPs. The in-situ generated outer CNT networks not only accommodated the huge changes in volume space inside Si nanoparticles but also provided fast electronic/ionic diffusion pathways, resulting in markedly improved cycling stability and rate ability. Furthermore, the facial and scalable preparation method created in this work could make this new Si-based anode material promising for practical application in next generation Li-ion batteries.

## Data Availability

The data presented in this study are available on request from the corresponding author.

## Supplementary Materials

The following are available online at https://www.mdpi.com/2079-4991/11/3/699/s1. Figure S1: XRD patterns of reduced Si NPs in the MR process, Si@RF, and Si@RF@SiO_2_. Figure S2: XPS spectrum of C1s (a) and O1s (b) of Si@C@v@CNTs. Figure S3: The pore size distribution curve of Si@C@v@C, Si@v@CNTs, and Si@C@v@CNTs. Figure S4: SEM images of Si@C@v@C. Figure S5: SEM images of Si@v@CNTs. Figure S6: TEM images of a single CNT. Figure S7: TEM images of Si@C@v@C. Figure S8: TEM images of Si@v@CNTs. Figure S9: The first five discharge-charge curves of Si@C@v@CNTs at current density of 100 mA g^−1^. Figure S10: Digital photograph (a), SEM image of Si@C@v@CNTs after the cycling test at 1.0 A g^−1^ (b,c). Figure S11: Charge/discharge profiles of NCM626 between 2.0–4.3 V. Figure S12: The electrochemical performance of the full cell using Si@C@v@CNTs as anode and LiNi_0.6_Co_0.2_Mn_0.2_O_2_ (NCM622) as cathode at the current density of 100 mA g^−1^. Table S1: The Brunauer-Emmett-Teller (BET) surface area, pore volume and average pore size of the samples.
