# Supplementary material for: Flexible Carbon Nanotubes Confined Yolk-Shelled Silicon-Based Anode with Superior Conductivity for Lithium Storage
Source: Nanomaterials (Basel). 2021 Mar 11;11(3):699. doi: 10.3390/nano11030699 (PMC8001621; doi:10.3390/nano11030699)
Supplement: Supplementary file 1 [file nanomaterials-11-00699-s001.pdf]

nanomaterials

Supplementary Materials

Flexible Carbon Nanotubes Confined Yolk-Shelled Silicon-Based Anode with Superior Conductivity for Lithium Storage

Na Han [1,†], Jianjiang Li [1,†], Xuechen Wang [1], Chuanlong Zhang [1], Gang Liu [1], Xiaohua Li [1], Jing Qu [1], Zhi Peng [1], Xiaoyi Zhu [1,*] and Lei Zhang [2,*]

[1] School of Material Science and Engineering, School of Environmental Science and Engineering, Chemical Experimental Teaching Center, School of Automation, Qingdao University, No. 308, Ningxia Road, Qingdao 266071, China; 2018020395@qdu.edu.cn (N.H.); jjli@qdu.edu.cn (J.L.); 2018020384@qdu.edu.cn (X.W.); 2018205858@qdu.edu.cn (C.Z.); 2019025785@qdu.edu.cn (G.L.); 2019020442@qdu.edu.cn (X.L.); 2017201339@qdu.edu.cn (J.Q.); pengzhi@qdu.edu.cn (Z.P.)

[2] Key Laboratory of Materials Physics, and Anhui Key Laboratory of Nanomaterials and Nanotechnology, Institute of Solid State Physics, Chinese Academy of Sciences, 230031, Hefei 230031, China

* Correspondence: xyzhu@qdu.edu.cn (X.Z.); lei.zhang@issp.ac.cn (L.Z.)

† These authors equally contributed to this work

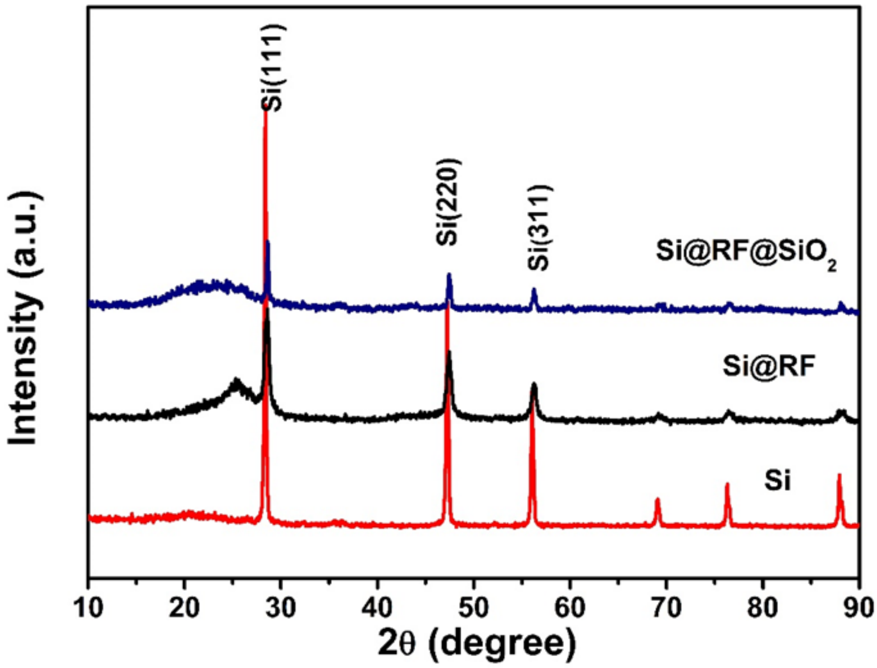

Figure S1. XRD patterns of reduced Si NPs in the MR process, Si@RF, and Si@RF@SiO_2.

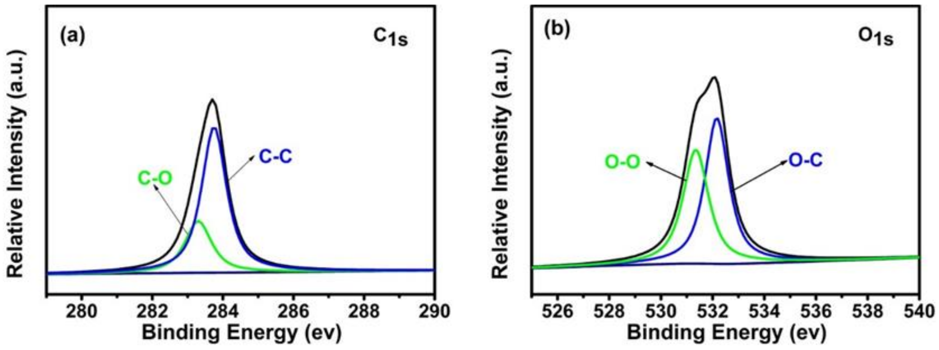

Figure S2. XPS spectrum of C_{1s} (a) and O_{1s} (b) of Si@C@v@CNTs.

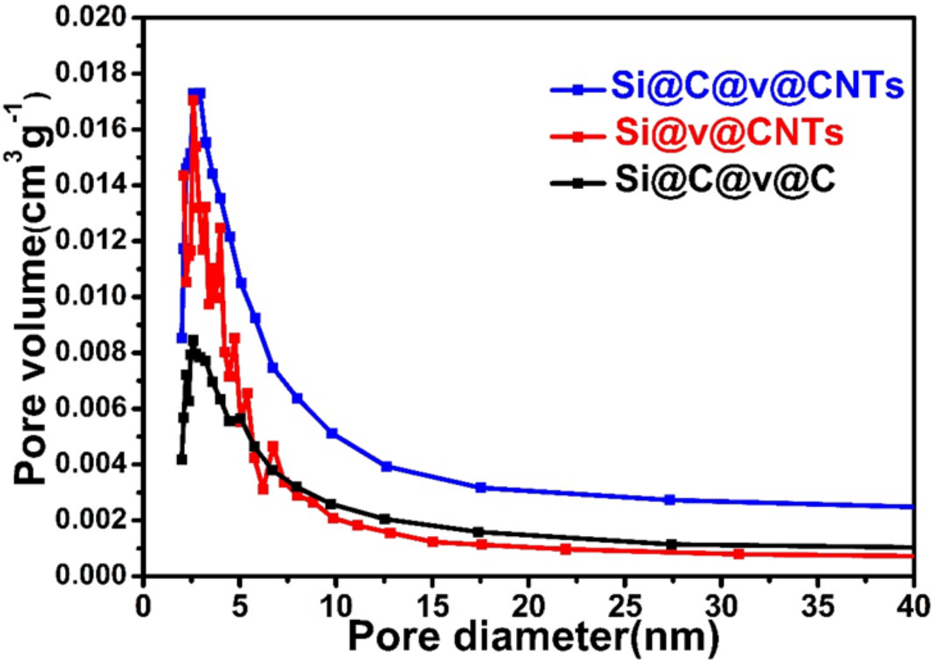

Figure S3. The pore size distribution curve of Si@C@v@C, Si@v@CNTs, and Si@C@v@CNTs.

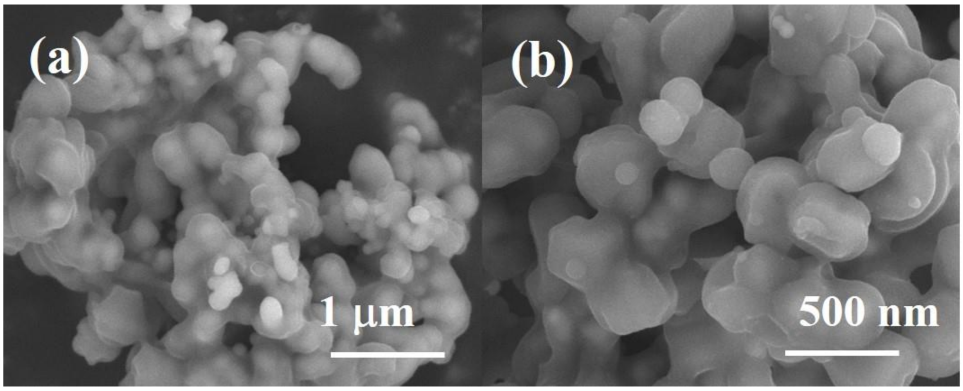

Figure S4. SEM images of Si@C@v@C (**a, b**).

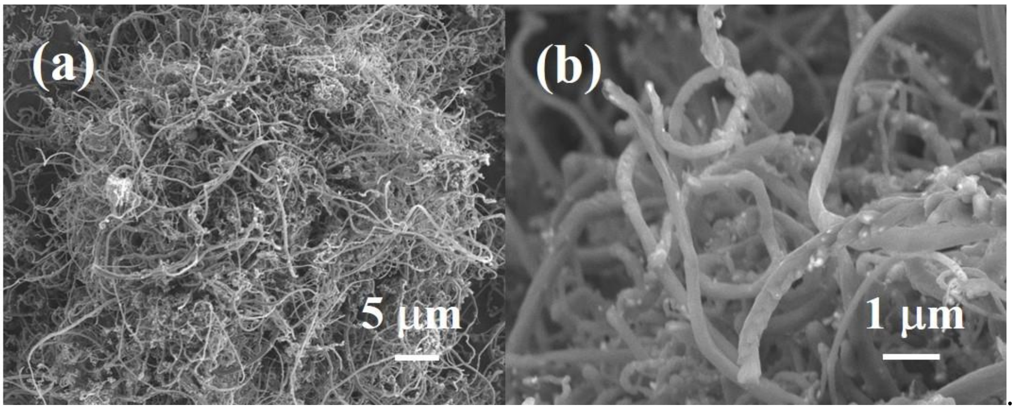

Figure S5. SEM images of Si@v@CNTs (**a, b**).

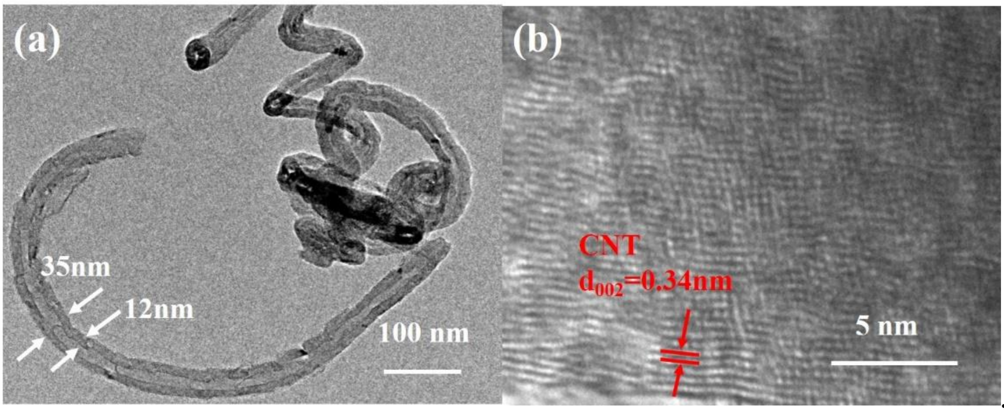

Figure S6. TEM images of a single CNT.

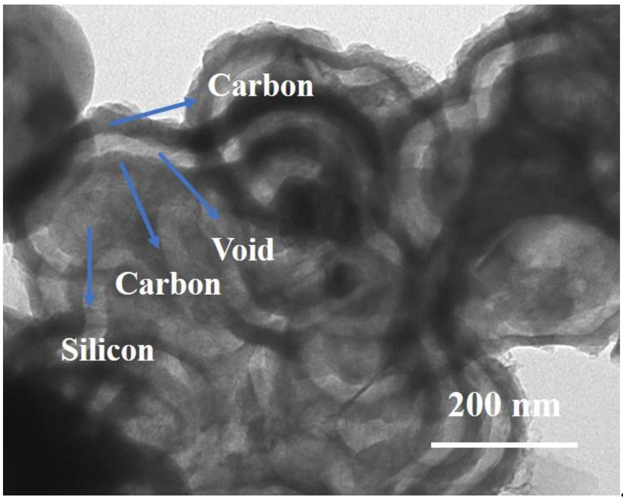

Figure S7. TEM images of Si@C@v@C.

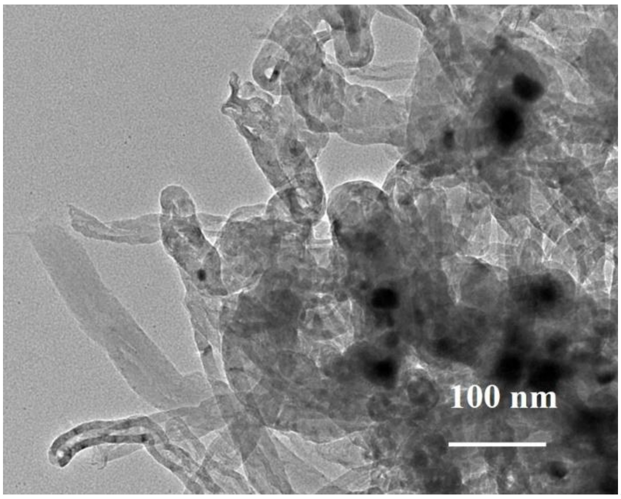

Figure S8. TEM images of Si@v@CNTs.

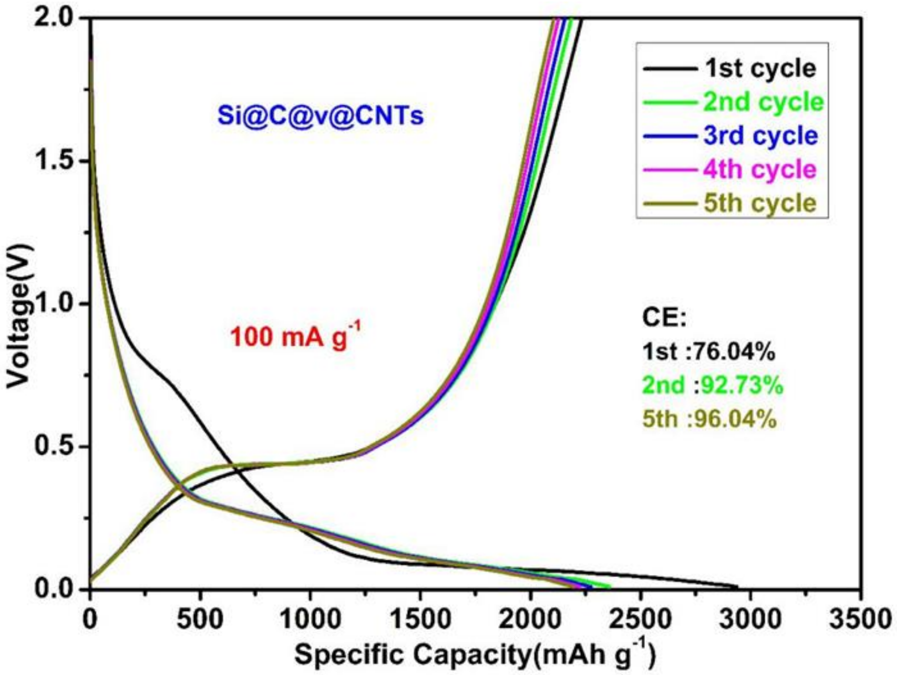

Figure S9. The first five discharge-charge curves of Si@C@v@CNTs at current density of 100 mA g^{-1}.

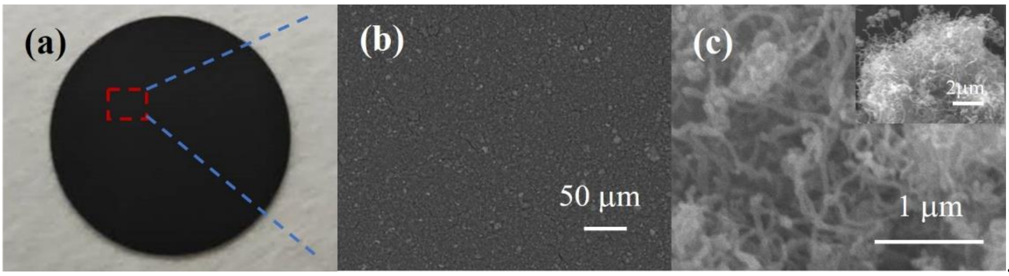

Figure S10. (**a**) Digital photograph, (**b, c**) SEM image of Si@C@v@CNTs after the cycling test at 1.0 A g^{-1}.

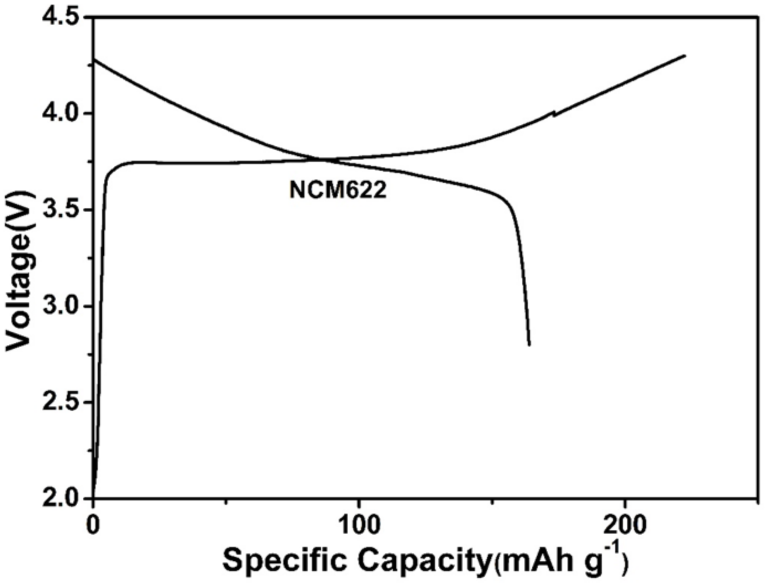

Figure S11. Charge/discharge profiles of NCM626 between 2.0–4.3 V.

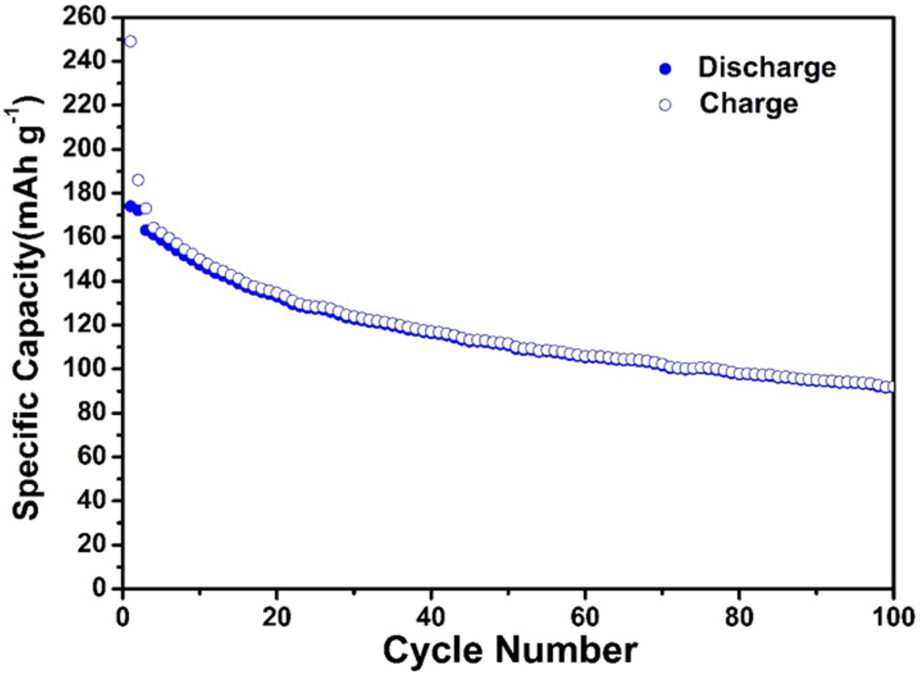

Figure S12. The electrochemical performance of the full cell using Si@C@v@CNTs as anode and $LiNi_{0.6}Co_{0.2}Mn_{0.2}O_2$ (NCM622) as cathode at the current density of 100 mA g^{-1}.

Table S1. The Brunauer-Emmett-Teller (BET) surface area, pore volume and average pore size of the samples.

Sample	Si@C@v@C	Si@v@CNT	Si@C@v@CNT
**Surface area (m^2 g^{-1})**	57.92	68.22	106.98
**Pore volume (cm^3 g^{-1})**	0.2127	0.1056	0.3212
**Average pore size (nm)**	24.5750	18.6534	21.0691
